# The LRRC8 volume‐regulated anion channel inhibitor, DCPIB, inhibits mitochondrial respiration independently of the channel

**DOI:** 10.14814/phy2.14303

**Published:** 2019-12-09

**Authors:** Aqeela Afzal, Eric E. Figueroa, Sujay V. Kharade, Kevin Bittman, Brittany K. Matlock, David K. Flaherty, Jerod S. Denton

**Affiliations:** ^1^ Department of Neurological Surgery Vanderbilt University Nashville Tennessee; ^2^ Department of Medicine Vanderbilt University Nashville Tennessee; ^3^ Department of Pharmacology Vanderbilt University Nashville Tennessee; ^4^ Department of Anesthesiology Vanderbilt University Medical Center Nashville Tennessee; ^5^ Agilent Technologies Santa Clara California; ^6^ Vanderbilt Vaccine Center Vanderbilt University Medical Center Nashville Tennessee

**Keywords:** DCPIB, LRRC8, mitochondria, respiration

## Abstract

There has been a resurgence of interest in the volume‐regulated anion channel (VRAC) since the recent cloning of the LRRC8A‐E gene family that encodes VRAC. The channel is a heteromer comprised of LRRC8A and at least one other family member; disruption of LRRC8A expression abolishes VRAC activity. The best‐in‐class VRAC inhibitor, DCPIB, suffers from off‐target activity toward several different channels and transporters. Considering that some anion channel inhibitors also suppress mitochondrial respiration, we systematically explored whether DCPIB inhibits respiration in wild type (WT) and LRRC8A‐knockout HAP‐1 and HEK‐293 cells. Knockout of LRRC8A had no apparent effects on cell morphology, proliferation rate, mitochondrial content, or expression of several mitochondrial genes in HAP‐1 cells. Addition of 10 µM DCPIB, a concentration typically used to inhibit VRAC, suppressed basal and ATP‐linked respiration in part through uncoupling the inner mitochondrial membrane (IMM) proton gradient and membrane potential. Additionally, DCPIB inhibits the activity of complex I, II, and III of the electron transport chain (ETC). Surprisingly, the effects of DCPIB on mitochondrial function are also observed in HAP‐1 and HEK‐293 cells which lack LRRC8A expression. Finally, we demonstrate that DCPIB activates ATP‐inhibitable potassium channels comprised of heterologously expressed Kir6.2 and SUR1 subunits. These data indicate that DCPIB suppresses mitochondrial respiration and ATP production by dissipating the mitochondrial membrane potential and inhibiting complexes I‐III of the ETC. They further justify the need for the development of sharper pharmacological tools for evaluating the integrative physiology and therapeutic potential of VRAC in human diseases.

## Graphical abstract

There has been a resurgence of interest in the volume‐regulated anion channel (VRAC) since the recent cloning of the LRRC8A‐E gene family that encodes VRAC. We demonstrate that the best‐in‐class VRAC inhibitor, DCPIB, inhibits mitochondrial respiration and ATP production by dissipating the mitochondrial membrane potential and inhibiting complexes I, II, and III of the electron transport chain. More specific pharmacological tools should be developed for exploring the integrative physiology and therapeutic potential of VRAC in human diseases.

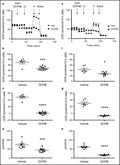

## 1 INTRODUCTION

Volume‐Regulated Anion Channels (VRAC) are ubiquitously expressed in mammalian cells where they play key roles in cell volume regulation and other fundamentally important cellular processes (Chen et al., 2019; Jentsch, 2016; Mongin, 2016; Osei‐Owusu, Yang, Vitery, & Qiu, 2018; Pedersen, Klausen, & Nilius, 2015; Stauber, 2015; Strange, Yamada, & Denton, 2019). Opening of VRACs by osmotic cell swelling allows chloride and small organic osmolytes (e.g., taurine, glutamate) to leave the cell, followed by osmotic water, to help restore cell volume and prevent membrane rupture. The discovery of VRAC more than 30 years ago (Cahalan & Lewis, 1988; Hazama & Okada, 1988) led to intensive investigation by numerous groups and an explosion in our understanding of their broad tissue distribution, the biophysical properties of the channels, and their regulation by diverse stimuli and cell signaling pathways (Chen et al., 2019; Jentsch, 2016; Jentsch, Lutter, Planells‐Cases, Ullrich, & Voss, 2016; Mongin, 2016; Osei‐Owusu et al., 2018; Pedersen et al., 2015; Stauber, 2015; Strange et al., 2019). However, the genes encoding the channels remained elusive and highly controversial until 2014 when two independent groups definitively identified the Leucine‐Rich Repeat Containing 8A‐E (LRRC8A‐E) genes that encode VRACs (Kumar et al., 2014; Voss et al., 2014) Results from gene disruption, heterologous expression, and cryo‐EM experiments indicate that VRACs are likely hexameric channels that contain LRRC8A and at least one other LRRC8 subunit (i.e., LRRC8B, LRRC8C, LRRC8D, or LRRC8E) (Strange et al., 2019). Importantly for the present study, several groups have shown that genetic disruption of the LRRC8A gene abolishes VRAC activity in a variety of different cell types (Bao et al., 2018; Platt et al., 2017; Stuhlmann, Planells‐Cases, & Jentsch, 2018; Trothe et al., 2018).

Since the discovery of the LRRC8 gene family, there has been a revitalization of interest in the physiological and pathophysiological roles VRAC plays in different cell types and tissues. By disrupting the expression of LRRC8A using knock‐down or knockout techniques, new roles of VRAC in drug uptake (Lambert & Sorensen, 2018), sperm development (Luck, Puchkov, Ullrich, & Jentsch, 2018), adipocyte biology (Zhang et al., 2017), pain (Wang et al., 2017), and insulin secretion (Stuhlmann et al., 2018) have recently been described. Mongin and colleagues demonstrated using siRNA against LRRC8A that VRACs are essential for swelling‐induced release of the excitatory amino acid glutamate in vitro (Hyzinski‐Garcia, Rudkouskaya, & Mongin, 2014; Schober, Wilson, & Mongin, 2017). This could have important clinical implications for the treatment of stroke since hypoxia leads to pathological cell swelling, activation of VRAC, and release of glutamate through VRAC into the brain. It was demonstrated recently that astrocyte‐specific knockout of LRRC8A reduces glutamate release and protects mice from excitotoxicity and brain damage following ischemic stroke (Yang et al., 2019).

Taken together, these data support the hypothesis that VRAC might represent a novel therapeutic target for stroke. In support of this notion, intracisternal administration of the VRAC inhibitor, DCPIB (4‐(2‐butyl‐6,7‐dichloro‐2‐cyclopentylindan‐1‐on‐5‐yl) oxybutyric acid), to rats prior to middle cerebral artery occlusion not only reduced infarct volumes, but also improved neurobehavioral scores 24 hr after stroke (Zhang, Zhang, Feustel, & Kimelberg, 2008). Unfortunately, DCPIB is unable to cross the blood–brain barrier when administered intravenously and suffers from poor selectivity (Zhang et al., 2008) DCPIB inhibits VRAC activity with an IC_50_ of approximately 5 µM, making it the most potent and best‐in‐class VRAC inhibitor currently available. However, at concentrations used to inhibit VRAC, DCPIB also inhibits H‐K‐ATPase (Fujii et al., 2015), inward rectifier potassium (Kir) channels (Deng, Mahajan, Baumgarten, & Logothetis, 2016), two pore‐domain potassium (K2P) channels (Lv et al., 2019), glutamate release via connexin hemichannels (Bowens, Dohare, Kuo, & Mongin, 2013), and glutamate uptake by GLT‐1 glutamate transporters (Bowens et al., 2013). Indeed, the molecular pharmacology of VRAC is plagued by weak and non‐selective inhibitors. For example, Niflumic acid and NPPB are not only weak blockers of VRAC, but they also have widespread off‐target effects including suppression of mitochondrial respiration (Ballatori, Truong, Jackson, Strange, & Boyer, 1995). The underlying mechanism is unknown but could reflect inhibition of mitochondrial chloride channels (O'Rourke, 2007) or non‐specific effects on the electron transport chain. Because it is increasingly appreciated that DCPIB is not as specific for VRAC as once believed, we set out to determine if DCPIB also inhibits mitochondrial respiration and, if so, whether this is dependent on VRAC function. Here, we report that DCPIB at concentrations used to inhibit VRAC suppresses mitochondrial respiration by uncoupling the mitochondrial membrane potential and inhibiting complexes I, II, and III of the electron transport chain (ETC). Surprisingly, these effects do not require the expression of LRRC8A and hence VRAC function.

## 2 MATERIALS AND METHODS

### 2.1 Chemicals

Rotenone, antimycin A, FCCP and oligomycin were purchased from Abcam. DCPIB (4‐(2‐butyl‐6,7‐dichloro‐2‐cyclopentylindan‐1‐on‐5‐yl) oxybutyric acid) was purchased from Tocris Bioscience. All other chemicals were purchased from Thermo Fisher Scientific unless otherwise noted.

### 2.2 HAP‐1 cell culture and genomic DNA sequencing

HAP‐1 wild type (WT) and LRRC8A‐knockout (KO) cells were purchased from Horizon Discovery and cultured at 37°C in a 5% CO_2_ incubator in Iscove's modified medium supplemented with 10% FBS. The cells were grown to 80% confluence for all experiments and used between passage 2 and 10. Both cells types were seeded on SensoPlates^TM^ (Greiner Bio‐One) and allowed to attach overnight. Genomic DNA was isolated from WT and LRRC8A‐KO HAP‐1 cells using a DNA miniprep kit, as per the manufacturer's protocols. The DNA was amplified with Titanium Taq (Takara Bio) using the following primers: LRRC8A Forward 5′‐GATCATCTTGTCTTGGGTGACCT‐3′, Reverse 5′‐ GGTATTTTGGACAATGGAAGAA‐3′. The amplified DNA was purified and sequenced using Sanger DNA sequencing (Genewiz).

### 2.3 RNA isolation and subunit quantification by qPCR

Total RNA was isolated from WT and LRRC8A‐KO HAP‐1 cells using the Aurum RNA isolation kit (BioRad). One microgram of RNA was reverse transcribed using Iscript (BioRad) and 100 ng of the resulting cDNA was used for real time PCR analysis. Gene specific, FAM labeled, Taqman probes for LRRC8A were used to amplify a single product, and the threshold cycle time (Ct) was determined. Relative RNA levels were calculated based on the Ct values and normalized the housekeeping gene, β‐actin.

### 2.4 Cell proliferation and morphology

WT and LRRC8A‐KO HAP‐1 cells were plated as described above. Following a 24‐hr incubation, cells were stained with 1 µg/ml Hoechst (Thermo Fisher Scientific), and the number of cells was quantified using the Lionheart Fx (BioTek). Cellular mitochondria were stained with 200 nM Mitotracker Deep Red (Thermo Fisher Scientific) for 20 min; cells were then fixed and permeabilized with 0.1% triton X and stained with Phalloidin for 30 min. Nuclei were counterstained with Hoechst, and cells were imaged at 60× under oil using the Lionheart Fx (BioTek).

### 2.5 Whole‐cell patch clamp electrophysiology

WT and LRRC8A‐KO HAP‐1 cells were cultured as described above. The day of experiments, the cells were rinsed with divalent‐free Hank's Balanced Salt Solution (HBSS), dissociated using 0.25% trypsin/1 mM EDTA, plated on poly(L‐lysine)‐coated round glass coverslips, and allowed to recover at 37°C in a 5% CO_2_ incubator for at least 1 hr before experiments. Micropipettes were pulled from Clark Custom 8,520 Patch Glass (1.5 o.d. × 1.16 i.d.) (Harvard Apparatus) using a P‐1000 Flaming/Brown Microelectrode puller (Sutter Instruments) to resistances of 2–4 MΩ when filled with the following solution (in mM): 116 NMDG‐Cl, 2 MgSO_4_, 20 HEPES, 6 CsOH, 1 EGTA, 2 Na_2_ATP, and 10 Sucrose (pH 7.2, 275 mOsm). The isotonic bath solution contained (in mM): 90 NMDG‐Cl, 50 MgSO_4_, 1 CaCl_2_, 12 HEPES, 8 Tris, 5 Glucose, 80 Sucrose, and 2 l‐glutamine (pH 7.4, 295 mOsm). The hypotonic bath solution contained (in mM): 90 NMDG‐Cl, 50 MgSO_4_, 1 CaCl_2_, 12 HEPES, 8 Tris, 5 glucose, and 2 L‐glutamine (pH 7.4, 225 mOsm). Cells were swollen by exposure to 225 mOsm bath solution. Osmolality was adjusted by the addition or removal of sucrose. Macroscopic currents were recorded under voltage‐clamp conditions using an Axopatch 200B amplifier (Molecular Devices, Sunnyvale, CA). Cells were voltage clamped at a holding potential of 0 mV and whole‐cell currents were elicited by voltage ramp or step protocols. For voltage ramps, the membrane potential was first stepped to −120 mV for 50 msec and then ramped over 1 s to +120 mV. This was followed by a step back to 0 mV for 4 s before ramp was repeated. Step changes in membrane voltage were induced by stepping membrane voltage to −120 mV to +120 mV in 800 msec, 20 mV increments. Data were collected at 5 kHz and filtered at 1 kHz. Data acquisition and analysis were performed using pClamp 9.2 software suite (Molecular Devices).

### 2.6 Seahorse respirometry assays

Cellular respirometry was performed using either a XF^e^24 (24‐well plate) or XF^e^96 (96‐well plate) extracellular flux analyzer (Agilent technologies). The XF^e^ flux analyzer measures the oxygen consumption rate (OCR) in monolayered cells using solid‐state sensor probes. These probes measure concentrations of dissolved oxygen every few seconds and calculate OCR from these measurements. Cell culture microplates (Agilent Technologies) were coated with 0.1 µg/ml poly‐L‐lysine, and plated with either 60,000 cells (XF^e^24) or 25,000 cells (XF^e^96). The cells were allowed to attach and grow for 24 hr in complete media supplemented with 10% FBS. Prior to respirometry analysis, complete media was replaced with assay media (modified DMEM supplemented with 10 mM glucose, 2 mM l‐glutamine, and 1 mM sodium pyruvate, pH 7.4), and the cells were placed in a CO_2_‐free incubator at 37°C for 1 hr to allow pH and temperature equilibration. Microplates were then loaded into the flux analyzer, equilibrated for 15 min, and subjected to three cycles of the following protocol: 3‐min mix, 2‐min wait, 3‐min read cycle (XF^e^24); or 3‐min mix followed by a 3‐min read cycle (XF^e^96). Serial injections of DCPIB (10 µM), oligomycin (1 µM, ATP synthase inhibitor), FCCP (1 µM, mitochondrial uncoupler), rotenone (0.5 µM, Complex I inhibitor), antimycin A (0.5 µM, Complex III inhibitor) were subsequently performed.

Measurement of real time mitochondrial ATP production was performed in a 24 well extracellular flux analyzer as described above. After cell plating and equilibration, serial injections of DCPIB (10 µM), oligomycin (1.5 µM) and rotenone/antimycin A (0.5 µM) were performed as described above. The real time ATP rate assay report generator (Agilent Technologies) was used to calculate mitochondrial ATP production.

All respirometry results were processed and analyzed using Wave software version 2.4. (Agilent Technologies). At the completion of each assay, cells were stained with 1 µg/ml Hoechst (Thermo Fisher Scientific) and the number of cells quantified using the Lionheart Fx (BioTek). All respirometry assays were normalized to cell counts in each well.

### 2.7 Quantitation of mitochondrial RNA and DNA

Total RNA was isolated and reverse transcribed from WT and LRRC8A‐KO HAP‐1 cells as described above. The resulting cDNA was used for real time PCR analysis. Gene specific, FAM labeled, Taqman probes for NADH‐ubiquinone oxidoreductase mitochondrial (NDUFS), succinate dehydrogenase (SDH), cytochrome B (CytB), cytochrome oxidase (CO), and mitochondrially encoded ATP synthase membrane subunit 6 (ATP6) were used to amplify a single product and determine the Ct values. Relative RNA levels were calculated based on the Ct values and normalized to beta actin. Genomic DNA (gDNA) was isolated from WT and LRRC8A‐KO HAP‐1 cells using a DNA miniprep kit, per the manufacturer's protocols (MilliporeSigma). The isolated gDNA was amplified using gene specific, FAM labeled Taqman probes for NADH‐ubiquinone oxidoreductase chain‐1 (ND‐1). Relative gDNA levels were calculated based on the Ct values and normalized to beta actin.

### 2.8 Mitochondrial uncoupling and membrane potential

Two assays were used to evaluate effects of DCPIB on mitochondrial membrane potential. In the first assay we substituted DCPIB for FCCP to assess whether DCPIB can uncouple oxidative phosphorylation and stimulate respiration. WT and LRRC8A‐KO HAP‐1 cells were seeded in an XF^e^24 plate as described above. Wells were sequentially injected with either oligomycin, FCCP, and rotenone/antimycin A (control) or with oligomycin, DCPIB (10 µM), and rotenone/antimycin A to assess oxygen consumption.

To assess mitochondrial membrane potential, 1 × 10^6^ cells were seeded in T25 flasks and allowed to attach. Cells were pretreated with FCCP (30 µM) or DCPIB (10 µM, 30 µM, or 100 µM) for 10 min and then stained with 5 nM tetramethylrhodamine, ethyl ester (TMRE) (Abcam) at 37°C, 5% CO_2_ for 15 min. Cells were washed, trypsinized, and analyzed on a BD LSRFortessa^TM^ flow cytometer (BD Biosciences).

### 2.9 Substrate oxidation by mitochondrial respiratory complexes

HAP‐1 cells were seeded onto XF^e^24 well plates and allowed to attach as described above. On the day of the assay, cells were washed with mitochondrial assay buffer (MAS: Sucrose (70 mM), mannitol (220 mM), potassium dihydrogen phosphate (5 mM), magnesium chloride (5 mM), HEPES (2 mM), EGTA (1 mM), bovine serum albumin (0.2%), adenosine diphosphate (4 mM), and XF plasma membrane permeabilizer (3 nM), pH 7.4). For assessment of each mitochondrial complex, different substrates (dissolved in MAS) were added to the wells (Table 1).

**Table 1:** ETC substrates and inhibitors used to isolate specific complexes

Complex	Substrates/inhibitors	Complex‐specific inhibitor
I	Pyruvate (10 mM)/malate (1 mM)	Rotenone (2 µM)
II	Succinate (10 mM)/rotenone (2 µM)	Antimycin A (2 µM)
III	Succinate (10 mM)/rotenone (2 µM)	Malonate (20 mM)
IV	Ascorbate (10 mM)/TMPD (100 µM)	Azide (20 µM)
V	Succinate (10 mM)	Oligomycin (2.5 µg/ml)

Microplates were then loaded into the flux analyzer and followed with two cycles of: 10‐s mix, 0‐min wait and a 2‐min read cycle. Serial injections of DCPIB (30 µM), Oligomycin (2.5 µg/ml), FCCP (4 µM) and rotenone/antimycin A (4 µM each) were performed for each cell type and respiratory control ratios (RCR) were derived by dividing state 3u respiration (FCCP induced maximal uncoupled stimulated respiration by state 4o respiration (Oligomycin induced inhibition of ATP synthase).

### 2.10 Thallium flux assays of K_ATP_ channel activity

Thallium flux assays of K_ATP_ channel activity were performed essentially as described previously (Raphemot et al., 2014). Briefly, stably transfected T‐REx‐HEK‐293 cells expressing Kir6.2/SUR1 were cultured in 384‐well plates (20,000 cells/20 *μ*L per well; black‐walled, clear‐bottomed PureCoat amine‐coated plates; BD bioscience) and cultured overnight with 1 μg/ml tetracycline to induce the expression of Kir6.2/SUR1. On the day of the experiment, the cell culture medium was replaced with dye‐loading solution containing assay buffer (HBSS with 20 mM HEPES, pH 7.3), 0.01% (v/v) Pluronic F‐127 (Life Technologies), and 1.2 μM of the thallium‐sensitive dye Thallos‐AM (WaveFront Biosciences). Following 1‐hr incubation at room temperature, the dye‐loading solution was washed from the plates and replaced with 20 μl/well of assay buffer. The plates were transferred to a Panoptic^®^ kinetic imaging plate reader (Wavefront Bioscience). A baseline recording was collected at 1 Hz for 10 s (excitation 470 ± 20 nm, emission 540 ± 30 nm) followed by addition of 20 *μ*L/well of test compounds dissolved in assay buffer. After 8‐min incubation period, 10 μl/well thallium stimulus buffer (125 mM NaHCO_3_, 1.8 mM CaSO_4_, 1 mM MgSO_4_, 5 mM glucose, 1.8 mM Tl_2_SO_4_, and 10 mM HEPES, pH 7.4) was added and data was collected for another 4 min. VU0071063 (Kharade et al., 2019; Raphemot et al., 2014) (positive control) and DCPIB were serially diluted threefold in DMSO and then dissolved in assay buffer to obtain 11‐point CRCs. The data acquisition and analysis were performed using Waveguide (WaveFront Biosciences) and Microsoft excel. EC_50_ values were determined by fitting the Hill equation using variable‐slope nonlinear regression analyses performed with GraphPad Prism version 7.02 (GraphPad Software).

### 2.11 Statistics

All data were plotted with GraphPad Prism version 7.03 (GraphPad Software). Data are depicted as mean ± *SEM*; to compare groups of values, Students *t‐test* was used; for mitochondrial membrane potential a one‐way ANOVA was used with post hoc Tukey's test. Significance is indicated as: *p ≤ .05; **p ≤ .01, ***p ≤ .001, and ****p ≤ .0001.

## 3 RESULTS

### 3.1 Swelling‐induced currents are absent in LRRC8A‐KO HAP‐1 cells

**Figure 1:**
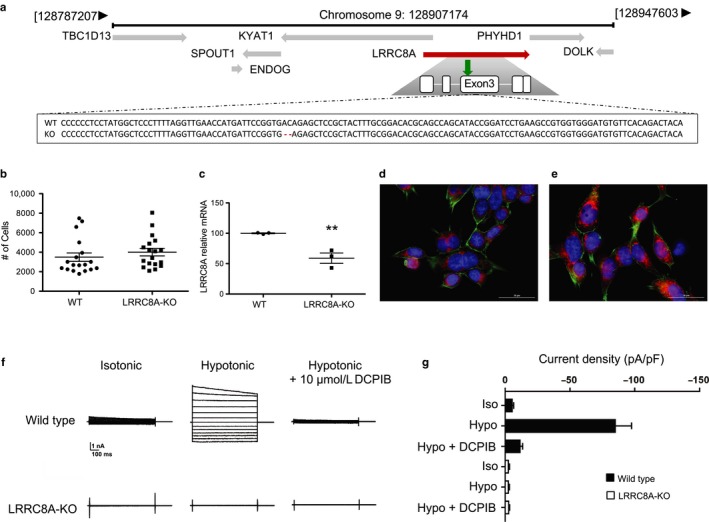
WT and LRRC8A‐KO HAP‐1 cell characterization. Sanger sequencing of WT and LRRC8A‐KO cells confirming location of deleted LRRC8A base pairs (a); surrounding genes include: tre‐2/usp6, BUB2, cdc16 domain family 13 (TBC1D13); Kynurenine aminotransferase 1 (KYAT1); phytanoyl‐CoA dioxigenase domain containing 1 (PHYHD1); SPOUT domain containing methyltrasnferase 1 (SPOUT1); dolichol kinase (DOLK); endonuclease G (ENDOG). Green arrow indicates location of the 2 base pair location (red dash) within exon 3 of the LRRC8A gene (indicated in red). Proliferation rates of both cell types (b) and gene expression of LRRC8A in WT and LRRC8A‐KO and (c). Graphs show mean ± *SEM* (*n* = 3); statistical analysis was carried out using student's *t*‐test. *Indicates *p* < .05. WT (d) and KO (e) cells stained with Mitotracker (red), phalloidin (green) and Hoechst (blue). Images are taken at 60×, scale bar = 30 microns. (f) Currents were measured by stepping V_m_ from −120 mV to +120 mV in 20‐mV increments. Cells were exposed to isotonic bath, hypotonic bath, or hypotonic bath +10 µM DCPIB to inhibit VRAC. (g) Mean ± *SEM* current density at −120 mV from WT and LRRC8A‐KO HAP1 cells measured using a ramp protocol from −120 mV and +120 mV under the indicated bath solution conditions (*n* = 5)

To evaluate the potential effects of DCPIB on mitochondrial respiration, we utilized commercially available human HAP‐1 cells in which the essential pore‐forming subunit of VRAC, LRRC8A, has been disrupted using CRISPR/Cas9 technology. Sanger sequencing of genomic DNA isolated from the HAP‐1 LRRC8A‐KO cells identified a two‐base‐pair deletion in exon 3 of LRRC8A (Figure 1a) that leads to a significant reduction in mRNA expression (Figure 1b). WT and LRRC8A‐KO HAP‐1 cells proliferate at similar rates (Figure 1c) and are morphologically similar (Figure 1d and e). Mitochondria exhibit a typical perinuclear distribution (Figure 1d and e) in both cell types.

To confirm that the two‐base‐pair deletion abolishes VRAC activity, we used whole‐cell patch clamp electrophysiology to measure the swelling‐induced anion current amplitude in HAP‐1 LRRC8A‐KO cells. As expected, WT cells exhibited robust swelling‐induced currents that were fully inhibited by 10 µM DCPIB. In striking contrast, HAP‐1 LRRC8A‐KO cells were completely devoid of VRAC activity. Thus, WT and LRRC8A‐KO HAP‐1 cells represent an appropriate cell model system for evaluating the VRAC‐independent effects of DCPIB on mitochondrial respiration (Figure 1f and g).

### 3.2 DCPIB inhibits mitochondrial respiration independently of LRRC8A‐dependent VRAC activity

We employed Seahorse respirometry to determine if DCPIB alters mitochondrial respiration in WT and LRRC8A‐KO HAP‐1 cells. The basic premise of this technology is as follows. Cells coordinate the Tricarboxylic Acid (TCA) cycle and oxidative phosphorylation to meet energy demands and generate respiratory intermediates necessary for cell growth and respiration. They take up glucose, convert it to pyruvate, which is transported to the mitochondria for oxidation under aerobic conditions. In the mitochondria, pyruvate is further converted into Acetyl CoA which enters the TCA cycle and generates high energy intermediates. These intermediates shuttle electrons through the ETC generating a proton gradient across the inner mitochondrial membrane. The mitochondrial stress tests used in this study employ pharmacological inhibitors of the ETC, proton gradient, or oxidative phosphorylation to determine if DCPIB alters OCR during respiration. Oligomycin, an ATP synthase inhibitor, hyperpolarizes the mitochondrial membrane potential, restricts protons from moving through the respiratory chain, and decreases OCR. The proton ionophore, FCCP, uncouples the proton gradient and forces the cells to recover the membrane potential by maximizing electron transport. This inhibitor increases OCR in Seahorse assays. Finally, the two electron chain inhibitors, antimycin A (complex II inhibitor) and rotenone (complex I inhibitor), inhibit mitochondrial respiration and reduces OCR. By measuring the effects of these inhibitors in the absence and presence of DCPIB, it is possible to determine if and how DCPIB alters mitochondrial respiration.

**Figure 2:**
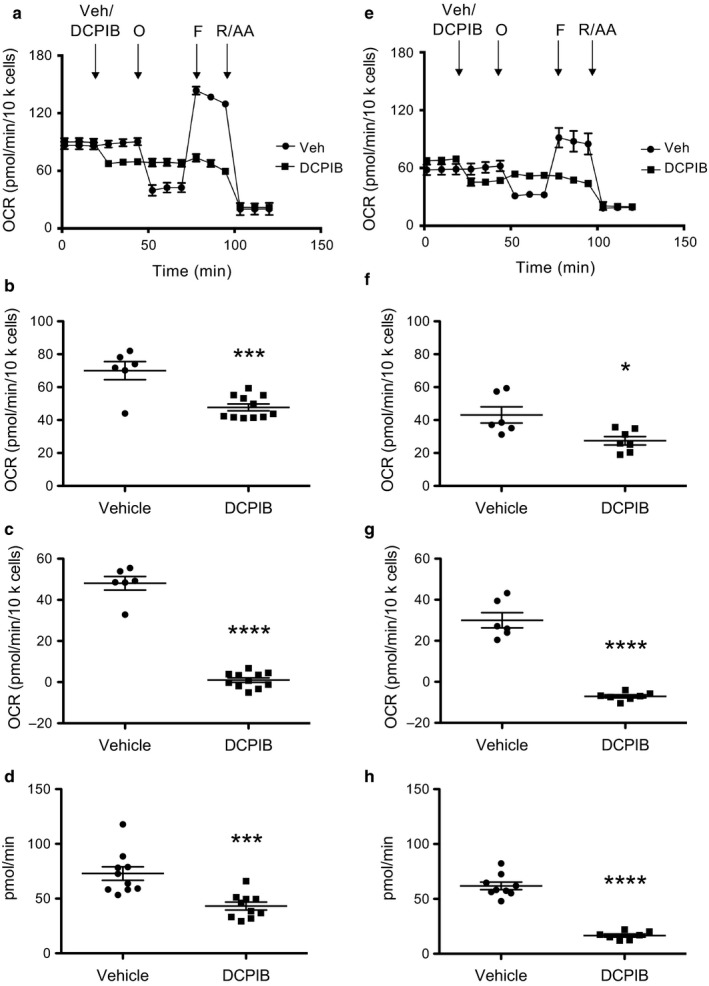
Mitochondrial respiration in WT and LRRC8A‐KO HAP‐1 cells. Mitochondrial respiration in presence of vehicle (circles) or DCPIB (squares) in WT cells (a) and LRRC8A‐KO cells (e). Basal respiration (WT cells (b) and LRRC8A‐KO cells (f) and ATP‐linked respiration (WT cells (c), LRRC8A‐KO cells (g) were calculated from the mitochondrial respiration graphs. ATP production in WT cells (d) and LRRC8A‐KO cells (h). Graphs show mean ± *SEM* (*n* = 3). Statistical analysis was carried out using student's *t*‐test; * indicates *p* < .05 compared to vehicle treated cells. O, Oigomycin; F, FCCP; R/AA, rotenone/antimycin A

The OCR of vehicle‐treated WT and LRRC8A‐KO cells was robust and predictably 1) reduced by the addition of oligomycin, 2) increased by FCCP, and 3) fully inhibited by co‐application antimycin A and rotenone (Figure 2)a and e). Addition of 10 µM DCPIB, a concentration that is commonly used to fully inhibit VRAC (e.g. Figure 1), led to a reduction in basal OCR (Figure 2)b and f) and ATP‐linked respiration (Figure 2c and g) in both cell types. Mitochondrial ATP production was also significantly lower in both cells types when treated with DCPIB (Figure 2d and h).

**Figure 3:**
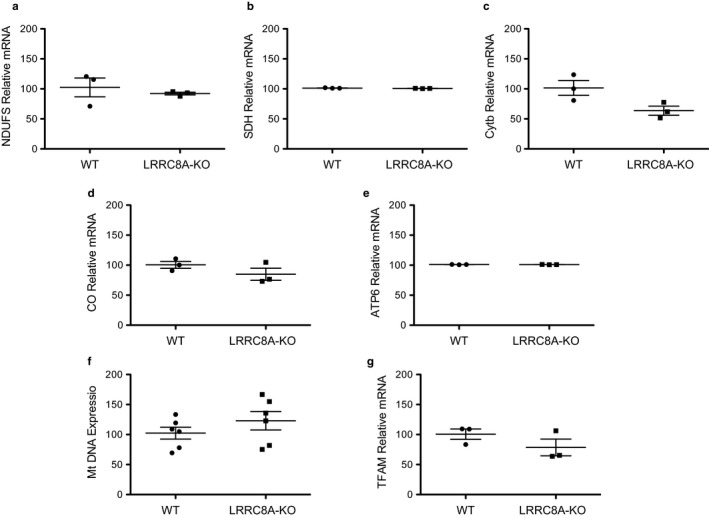
Mitochondrial gene expression is not different between WT and LRRC8A‐KO HAP‐1 cells. NADH‐ubiquinone oxidoreductase (NDUFS) complex I (a), Succinate dehydrogenase (SDH) complex II (b), Cytochrome b (cytb) Complex III (c), Cytochrome Oxidase (MT‐CO1) complex IV (d), and mitochondrial encoded ATP synthase membrane subunit 6 (MT‐ATP6) complex V (e) levels in WT and LRRC8A‐KO cells were not significantly different. Mitochondrial DNA (f) and mitochondrial transcription factor A (TFAM) expression (g) were also not significantly different between the two groups (*n* = 3)

To determine if knocking out LRRC8A has direct effects on mitochondrial biology, we assessed mitochondrial complex gene expression, mitochondrial mass (mitochondrial DNA expression), and mitochondrial transcriptional factor A. No significant differences were observed between the WT and LRRC8A‐KO cells (Figure 3).

**Figure 4:**
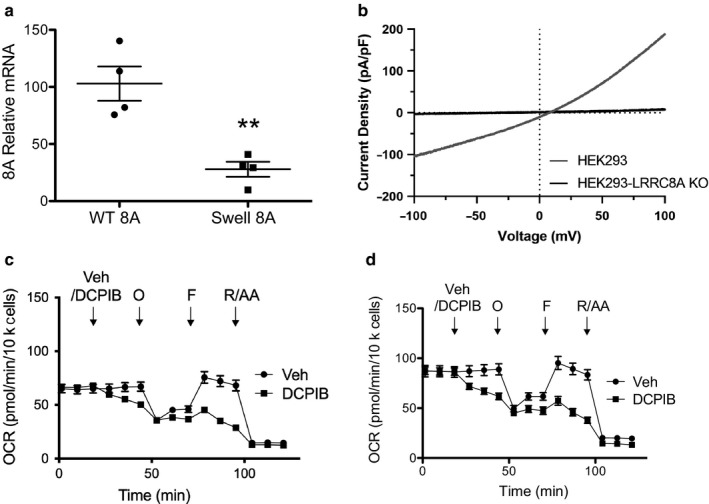
DCPIB alters mitochondrial respiration in HEK‐293 cells. (a) Gene expression of LRRC8A in WT and LRRC8A KO cells (*n* = 4). (b) Currents were measured by stepping V_m_ from −200 mV to +200 mV in 20‐mV increments. Cells were exposed to isotonic bath, hypotonic bath, or hypotonic bath +10 µM DCPIB to inhibit VRAC. Mitochondrial respiration in presence of vehicle (circles) or DCPIB (10 µM, squares) in (c) WT and (d) LRRC8A‐KO HEK cells. Mitochondrial respiration was measured by sequential addition of vehicle/DCPIB (10 µM), oligomycin (1 µM), FCCP (1 µM) and rotenone & antimycin A (0.5 µM each). O, oligomycin; F, FCCP; R/AA, rotenone/antimycin A

To ensure that the effects of DCPIB on mitochondrial respiration are not unique to HAP‐1 cells, we performed the same stress tests on WT and LRRC8A‐KO HEK‐293 cells (Figure 4). LRRC8A‐KO HEK‐293 cells had reduced expression of LRRC8A mRNA (Figure 4a) and exhibited no swelling‐induced anion currents (Figure 4b). As we observed in HAP‐1 cells, DCPIB altered the mitochondrial responses to the panel of inhibitors in both WT and LRRC8A‐KO HEK‐293 cells (Figure 4c and d), confirming the effects are not unique to HAP‐1 cells and are independent of LRRC8A‐dependent VRAC function.

### 3.3 DCPIB uncouples oxidative phosphorylation and lowers mitochondrial membrane potential

**Figure 5:**
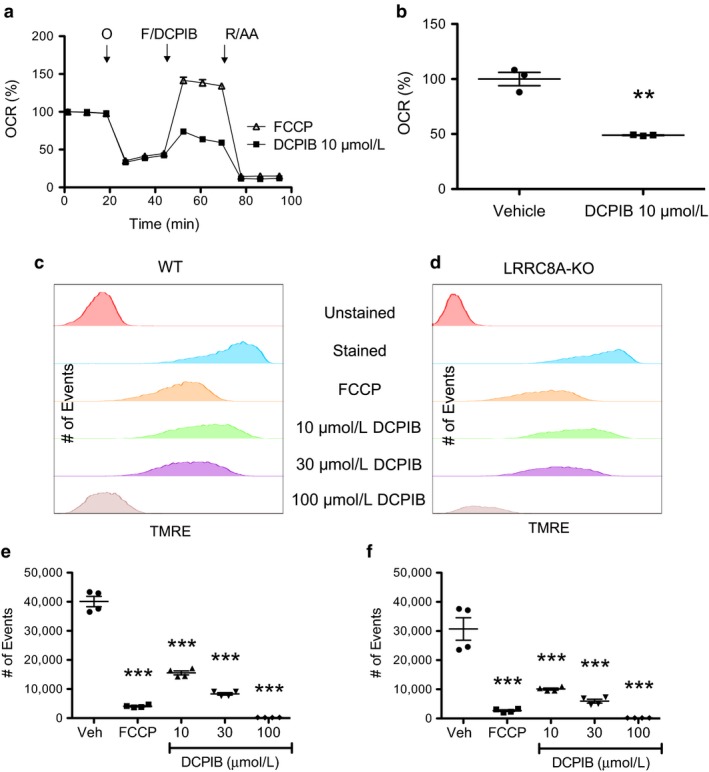
DCPIB acts as a mitochondrial uncoupler. WT HAP‐1 cells were subjected to a standard mitochondrial stress test (a) with sequential injections of oligomycin, FCCP, rotenone & antimycin A (circles) or oligomycin, DCPIB (10 µM), rotenone & antimycin A (squares). Maximal uncoupled respiration of vehicle and DCPIB‐treated cells (b) shows that DCPIB uncouples mitochondrial respiration. Graph shows mean ± *SEM* (*n* = 3). Statistical analysis was carried out using student's *t*‐test *indicates *p* < .05 compared to vehicle. Histogram of mitochondrial membrane potential in (c) WT and (d) LRRC8A‐KO HAP‐1 cells using TMRE. Cells were exposed to either FCCP (30 µM), or DCPIB at 10, 30 and 100 µM. Graph shows mean ± *SEM* (*n* = 4). Mean ± *SEM* TMRE fluorescence of (e) WT or (f) LRRC8A‐KO cells measured with flow cytometry. Statistical analysis was carried out using one‐way ANOVA and Tukey's post hoc analysis. *indicates *p* < .05 compared to vehicle. O, Oligomycin; F, FCCP; R/AA, rotenone/antimycin A

Considering the altered profile of mitochondrial respiration in response to DCPIB, we investigated whether DCPIB directly uncouples the mitochondrial proton gradient and depolarizes the membrane potential in HAP‐1 cells. As expected, following oligomycin addition, FCCP increased OCR by uncoupling the mitochondrial proton gradient and enhancing electron transport (Figure 5a). Similarly, addition of 10 µM DCPIB also enhanced OCR, albeit to a lesser degree than FCCP (Figure 5b). We next used the cell permeant dye, TMRE, and flow cytometry to test if DCPIB depolarizes the mitochondrial membrane potential. TMRE accumulates in active mitochondria with hyperpolarized membrane potentials but accumulates to a lesser degree in depolarized mitochondria. Thus, TMRE fluorescence intensity can be used as a proxy measurement of mitochondrial membrane potential. Treatment of WT and LRRC8A‐KO HAP‐1 cells with FCCP resulted in a significant decrease in TMRE fluorescence, as expected for cells with uncoupled mitochondrial respiration (Figure 5c–f). Treatment of both cell types with DCPIB led to a dose‐dependent and significant reduction in TMRE fluorescence, confirming that DCPIB depolarizes the mitochondrial membrane potential independently of LRRC8A‐dependent VRAC function.

### 3.4 DCPIB has widespread effects on the mitochondrial respiratory chain

**Figure 6:**
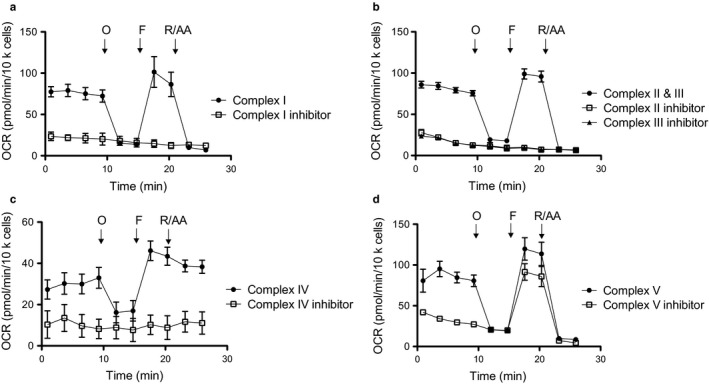
Pharmacological isolation of mitochondrial complexes in respirometry assays. HAP‐1 cells were permeabilized, treated with ETC complex‐specific substrates in the absence or presence of inhibitors, and then subjected to oligomycin (O), FCCP (F), and rotenone/antimycin A (R/AA) to assess respiration at each complex. (a) Complex 1 substrates were pyruvate (10 mM) and malate (1 mM). Complex I inhibitor was rotenone (2 µM). (b) Complexes II and III were isolated using the substrate succinate (10 mM) and Complex I inhibitor, rotenone (2 µM). Complex II was inhibited with antimycin (2 µM), whereas Complex III was inhibited with malonate (20 mM). (c) Complex IV was stimulated using ascorbate (10 mM) and N1, N1, N1, N1‐tetramethyl‐1,4‐phenylene diamine (100 µM) and inhibited using azide (20 mM). (d) Succinate (10 mM) was used to stimulate Complex V‐linked respiration, and oligomycin (2.5 µg/ml) was used to inhibit Complex V. Respiratory control ratios (RCR) were calculated by dividing state 3u by state 4o respiration (*n* = 4)

**Figure 7:**
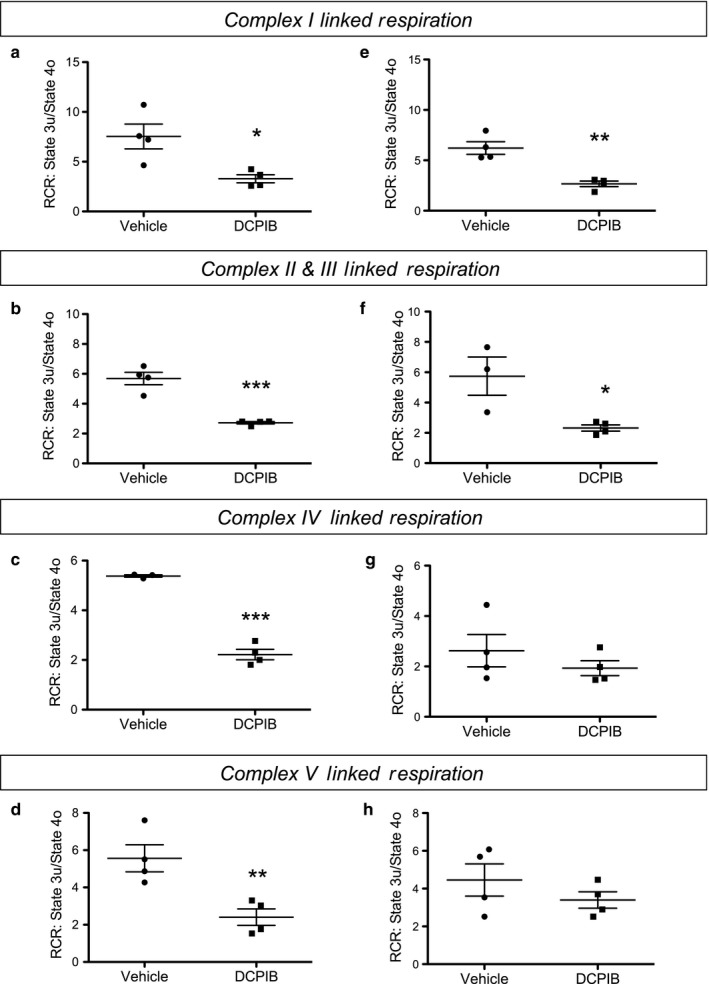
Mechanisms of altered mitochondrial respiration in response to DCPIB. ETC complex isolation experiments were performed in WT (left column) and LRRC8A‐KO (right column) HAP‐1 cells with conditions as described in Figure 6 and Table 1. DCPIB significantly inhibits Complex I in (a) WT and LRRC8A‐KO cells, Complex II/III in (c) WT and (d), and Complex IV in (e) WT cells. Graphs show mean ± *SEM* (*n* = 4). Statistical analysis was carried out using student's *t*‐test. * Indicates *p* < .05

To further explore the molecular mechanisms by which DCPIB inhibits mitochondrial function, we developed Seahorse assays for HAP‐1 cells to measure the activity of complexes I, II/ III, IV, and V of the ETC. These assays were adapted from the methods described by Divakaruni et al. 2014 (Divakaruni, Rogers, & Murphy, 2014). Briefly, following permeabilization of the cell membrane to allow complex‐specific substrates to be added to mitochondria, OCR is measured in the absence or presence of complex inhibitors to ensure specificity (Table 1). As shown in Figure 6a–d, robust substrate‐dependent OCR for each complex could be measured in vehicle‐treated cells and inhibited with complex‐specific inhibitors. After establishing these assays, we next tested if 30 µM DCPIB inhibited any of the complexes along the ETC. As shown in Figure 7a–f, DCPIB led to a significant reduction in complex I and II/III activity in both WT and LRRC8A‐KO HAP‐1 cells. DCPIB also inhibited complex IV‐ and V‐linked respiration in WT cells, but this significant effect was lost in the LRRC8A‐KO cells.

### 3.5 DCPIB activates ATP‐inhibitable Kir6.2/SUR1 potassium channels

**Figure 8:**
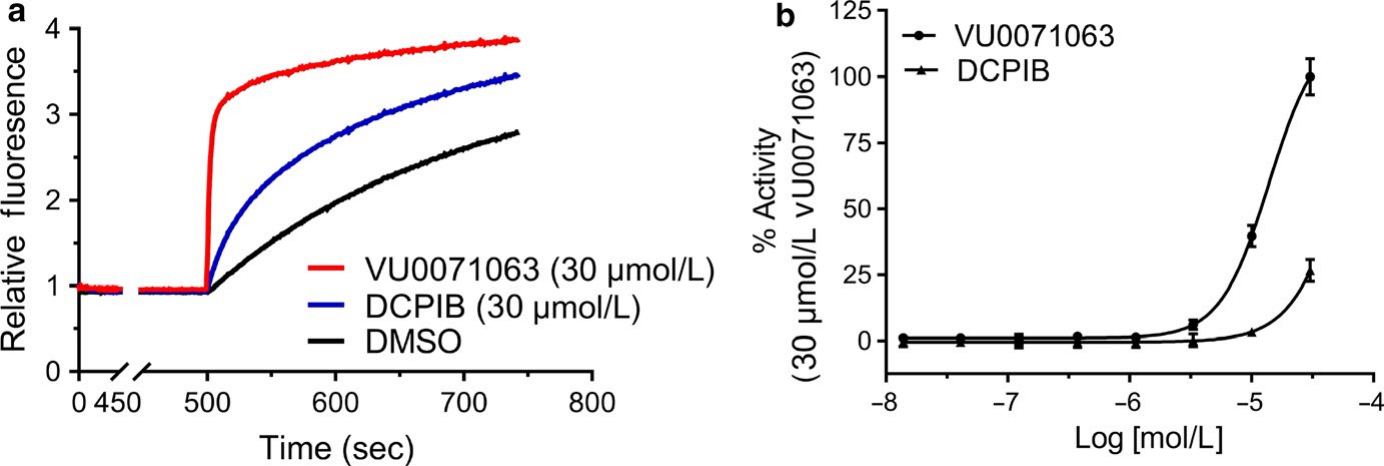
Activation of Kir6.2/SUR1 channels by DCPIB in thallium flux assays. (a) Representative traces showing increase in thallium induced fluorescence overtime in wells treated with 30 µM concentration of either VU0071063 (positive control), DCPIB, or DMSO (vehicle control). (b) Concentration‐response curves showing dose‐dependent activation of Kir6.2/SUR1 channels expressed in HEK‐293 cells in response to VU0071063 (specific Kir6.2/SUR1 channels activator) and DCPIB. (b) Representative traces showing increase in thallium‐induced fluorescence overtime in wells treated with 30 µM concentration of either VU0071063 (positive Control), DCPIB, or DMSO (vehicle control)

The data presented above are consistent with the notion that DCPIB inhibits mitochondrial respiration and ATP production independently of LRRC8A‐dependent VRAC function. As an orthogonal assay of DCPIB‐dependent inhibition of ATP synthesis, we tested whether DCPIB could activate potassium channels comprised of the inward rectifier potassium (Kir) channel pore forming subunit, Kir6.2, and the regulatory sulfonylurea receptor (SUR) subunit SUR1. Kir6.2/SUR1 channels are ATP‐gated potassium channels expressed predominantly in pancreatic beta cells and neurons in the brain where they serve to couple metabolic status, via ATP‐dependent channel inhibition, to cell excitability (Nichols, 2006; Nichols, Enkvetchakul, & Flagg, 2006). We reasoned that if DCPIB does indeed inhibit ATP synthesis, then it should lead to the activation of Kir6.2/SUR1. Channel activity was measured using a thallium flux assay that reports the inwardly directed movement of thallium into T‐REx‐HEK‐293 cells heterologously expressing Kir6.2/SUR1 (Kharade, Nichols, & Denton, 2016). As shown in Figure 8, treatment of T‐REx‐HEK‐293‐Kir6.2/SUR1 cells with the SUR1‐specific activator, VU0071063 (Kharade et al., 2019; Raphemot et al., 2014) (positive control), or DCPIB, led to an increase in thallium flux in HEK‐293‐Kir6.2/SUR1 cells. Taken together, these data indicate that DCPIB suppresses mitochondrial respiration and ATP synthesis in HAP‐1 and HEK‐293 cells independently of LRRC8A‐dependent VRAC.

## 4 DISCUSSION

The major finding of this study is that the best‐in‐class VRAC inhibitor, DCPIB, suppresses ATP production via uncoupling of the mitochondrial membrane potential and inhibition of complexes I, II, and III of the respiratory chain at concentrations typically used to inhibit VRAC. Thus, mitochondria can be added to the growing list of DCPIB off‐targets that includes H‐K‐ATPase (Fujii et al., 2015), Kir channels (Deng et al., 2016), K2P channels (Vivier, Bennis, Lesage, & Ducki, 2016), connexin hemi‐channels (Ye, Oberheim, Kettenmann, & Ransom, 2009), and glutamate transporters (Bowens et al., 2013). At a time when there is growing interest in VRAC integrative physiology due to the recent cloning of the LRRC8 gene family (Kasuya et al., 2018; Kefauver et al., ,; Syeda et al., 2016; Yamada & Strange, 2018), investigators are faced with a paucity of small‐molecule inhibitors that can be used to specifically modulate the channel in vitro and in vivo. While some experiments investigating VRAC function/physiology might not be sensitive to inhibition of certain pumps, channels, or transporters, or in whole‐cell patch clamp experiments where ATP is included in the pipette solution, inhibition of ATP production by DCPIB in intact cells will have widespread, confounding effects on many different aspects of cell, tissue, and organismal physiology. Caution should be used when designing experiments that employ DCPIB as a specific inhibitor of VRAC.

In principle, DCPIB could suppress mitochondrial respiration by (a) inhibiting pyruvate synthesis in the cytoplasm and/or transport of pyruvate across the inner mitochondrial membrane, (b) dissipating the IMM potential, or (c) directly inhibiting complexes of the ETC. We believe it is unlikely that DCPIB disrupts pyruvate availability for the following reason. Pyruvate is the final product of aerobic glycolysis and must be transported from the cytoplasm across the inner mitochondrial membrane (IMM) and into the mitochondrial matrix where it is converted to acetyl CoA, a major fuel for the ETC. In the mitochondrial stress tests used in this study, pyruvate was provided as an exogenous energy sources to drive oxidative phosphorylation and oxygen consumption. Therefore, inhibition of glycolytic enzyme activity and consequently new pyruvate synthesis by DCPIB should have no effects on OCR in these assays. We cannot rule out whether DCPIB inhibits pyruvate transport into the mitochondrial matrix.

We found that DCPIB is a mitochondrial uncoupler. The voltage across the IMM is generated when electron equivalents are transported along the ETC and protons are pumped across the IMM and into the intermembrane space. The energy contained within the proton gradient is used to drive the synthesis of ATP by complex V (ATP synthase). Mitochondrial uncouplers such as FCCP create pores in the IMM that allow protons to escape down their electrochemical gradient and back into the mitochondrial matrix. In Seahorse assays, collapse of the proton gradient by FCCP leads to an increase in OCR as oxygen consumption by complex IV reaches a maximum. When administered instead of FCCP, 10 µM DCPIB increases OCR in a fashion similar to that of FCCP. In the presence of DCPIB, the increase in OCR induced by FCCP is abolished, suggesting the two compounds acted on the same molecular target or pathway.

The membrane potential across the IMM can be monitored using the cationic dye, TMRE, which accumulates in mitochondria due to strong negative voltage (i.e., −180 mV) across the IMM. Dissipation of the proton gradient by chemical uncouplers such as FCCP causes depolarization of the mitochondrial membrane potential and reduction in TMRE fluorescence. In this study, we found that DCPIB led to a dose‐dependent decrease in TMRE fluorescence. These data suggest that DCPIB inhibits mitochondrial respiration, at least in part, by dissipating the proton gradient across the IMM.

We evaluated the sensitivity of complexes I‐V in the ETC to 30 μM DCPIB. In these assays, a threefold‐higher drug concentration was used to compensate for the use of BSA and consequent DCPIB binding to serum protein. The plasma membrane was permeabilized using a propriety solution to enable the delivery of solutions containing combinations of complex‐specific substrates and inhibitors to isolate the contributions of individual complexes to the OCR. The IMM does not appear to be permeabilized using this solution (Divakaruni et al., 2014; Salabei, Gibb, & Hill, 2014). We found that DCPIB inhibits the OCR mediated by complexes I (NADH dehydrogenase), II (succinate dehydrogenase), and III (cytochrome bc1 complex), with a trend toward inhibition of complexes IV (cytochrome c oxidase) and V (ATP synthetase). Because the IMM is not permeabilized in these assays, we cannot rule out the possibility that DCPIB inhibits transport of complex substrates across the IMM. However, we feel the simplest explanation is that DCPIB inhibits the activities of complexes I‐III.

Considering that DCPIB is known to have off‐target effects on other ion channels (see below), it is conceivable that suppression of respiration is related to inhibition of ion channels present in mitochondria. Indeed, several different types of anion and cation channels are known to be expressed in mitochondria. These include the voltage‐dependent anion channel (VDAC), KCa1.1, KCa3.1, KCa2.x, Kv1.3, Kv1.5, Kv7.4, Kir1.1b, K_ATP_, and K2P9.1 (TASK‐3) (Leanza et al., 2018). Interestingly, DCPIB has been shown to inhibit TASK‐3 and K_ATP_ channels. However, it is unclear if the effects of DCPIB on mitochondrial respiration described in the present study are due to inhibition of these channels or other molecular targets.

The inhibitory effects of DCPIB on K_ATP_ noted above may seem contradictory to our data showing that DCPIB activates, not inhibits, K_ATP_. The assay used here is based on the heterologously expressed pancreatic/brain K_ATP_ channel subtype comprised of Kir6.2 and SUR1, which is inhibited by intracellular ATP (Aguilar‐Bryan et al., 1995; Inagaki et al., 1995). We used the channel as a biosensor of intracellular ATP concentrations to test if DCPIB activates Kir6.2/SUR1 as a consequence of intracellular ATP depletion. Although we did observe channel activation, this required a concentration of 30 μM DCPIB, which is higher than the dose used in the Seahorse assays. Logothetis and colleagues recently reported that DCPIB inhibits Kir6.2/SUR1 channels expressed in *Xenopus laevis* oocytes (Deng et al., 2016). Inhibition was also observed in a Kir6.2 C‐terminal truncation mutant that allows the channel to be expressed in the absence of SUR1, indicating that the DCPIB binding site is located in the pore‐forming Kir6.2 subunit. Taken together with our data, the apparently weak activation of Kir6.2/SUR1 observed in the present study might reflect a combination of Kir6.2/SUR1 activation and inhibition by DCPIB.

It is important to note that in cells lacking LRRC8A expression, the remaining LRRC8B‐E subunit are still expressed (Voss et al., 2014). It is conceivable, albeit unlikely given what is currently known about the requirement of LRRC8A for the assembly of functional VRAC channels (Qiu et al., 2014; Voss et al., 2014), that other LRRC8 subunits form DCPIB receptors in mitochondria. It will be important in future studies to determine if different LRRC8 subunits are expressed in mitochondria and contribute to respiration.

In conclusion, we have shown that the current best‐in‐class inhibitor of VRAC, DCPIB, suppresses mitochondrial respiration and ATP production by uncoupling the mitochondrial proton gradient and inhibiting complexes I, II, and III of the ETC. Because these effects are observed in cells lacking the expression of the essential VRAC subunit, LRRC8A, they are likely unrelated to inhibition of VRAC channel function. This study emphasizes the need for improved pharmacological tools to investigate the integrative physiology of the channel where metabolism could be an important factor. We recently reported the discovery of two CysLT1 receptor antagonists, Pranlukast and Zafirlukast, as novel‐scaffold inhibitors of VRAC (Figueroa, Kramer, Strange, & Denton, 2019). These compounds could be used as starting points in lead optimization with medicinal chemistry to develop analogs with improved potency and specificity for VRAC. In light of the data presented here, it will be important to evaluate lead compounds for activity toward mitochondrial respiration.

## AUTHOR CONTRIBUTIONS

AA, EF, SVK, JSD, BKM, DKF and KB contributed to research design. AA, EF, BKM, DKF and SVK conducted experiments. AA, EF, and SVK performed data analysis. AA, EF, SVK, and JSD wrote manuscript.
